# Current Assistive Devices Usage and Recommendations for a Future Artificial Vision Prosthesis among Patients with Severe Visual Impairment Due to Inherited Retinal Diseases

**DOI:** 10.3390/jcm12165283

**Published:** 2023-08-14

**Authors:** Sophia Sidhu, Patrice J. Persad, Byron L. Lam, Kasey L. Zann, Ninel Z. Gregori

**Affiliations:** 1 Department of Ophthalmology, Bascom Palmer Eye Institute, University of Miami Miller School of Medicine, Miami, FL 33136, USA; sdsidhu@health.ucsd.edu (S.S.); p.persad@med.miami.edu (P.J.P.); blam@med.miami.edu (B.L.L.); 2 UC San Diego School of Medicine, San Diego, CA 92093, USA; 3 Miami Veterans Affairs Medical Center, Miami, FL 33125, USA; kasey.zann@va.gov

**Keywords:** Argus II prosthesis, artificial vision, assistive devices, blindness, inherited retinal diseases, retinal prosthesis, visual impairment

## Abstract

Patients with inherited retinal diseases (IRDs) utilize various adaptive techniques and devices designed to assist them with activities of daily living (ADLs). The purpose of this study was to assess the assistive devices used by patients with IRDs, the difficulties they face despite these devices, and their recommendations for a future visual prosthesis. In collaboration with blind patients, an online survey was developed and administered to adults with IRDs and visual acuities of 20/400 to no light perception in the better-seeing eye. We analyzed data from 121 survey respondents (aged 18 to >80 years). Five respondents were Argus II prosthesis recipients. The most commonly used aids were cellular phones/tablets for reading (63.6%) as well as a sighted guide (75.0%) and a cane (71.4%) for mobility. Despite current assistive devices, participants reported continued difficulty with ADLs. Improved navigation, reading, and facial recognition were ranked the most desirable features for future visual prostheses. Argus II recipients suggested technology with improved ability to recognize objects and obstacles, detect movement, and cut out busy backgrounds. These insights are valuable in shaping the design of future prosthetic devices tailored to the needs of IRD patients.

## 1. Introduction

Inherited retinal diseases (IRDs) encompass a group of ocular conditions that can cause severe vision impairment and profound blindness through the progressive loss of retinal cells [1], with over 5.5 million people affected worldwide [1,2]. 

Patients with IRDs utilize various adaptive techniques and devices designed to assist them with activities of daily living (ADLs). Implantation of a retinal prosthetic that produces artificial vision and restores some degree of vision represents an innovative therapy for treating patients with IRDs [3,4,5,6,7,8]. Since 2013, over 300 patients worldwide with visual acuities (VAs) of bare light perception or no light perception in both eyes due to end-stage retinitis pigmentosa and other outer retinal degenerations received the Argus^®^ II Retinal prosthesis, which consists of an episcleral implant connected to a 60- microelectrode array attached to the inner surface of the retina [5,6,7,8]. Newer, potentially higher resolution retinal prosthetic devices and other novel approaches such as optogenetics via gene therapy [9] and visual cortex prosthesis [10] are currently under development. 

Although several studies have assessed the patient-reported outcomes and quality of life in patients with IRDs [11,12], more research is needed to assess which activities patients with various end-stage IRDs rank as most significant in their lives and which ones they would like to improve with future assistive devices. The insights and recommendations of these patients are integral to informing the design of future prosthetic devices that can better target their daily activity needs.

To achieve this goal, we designed and administered an anonymous online survey to patients with VAs of 20/400 to no light perception in the better-seeing eye due to various IRDs. The aim was to assess the assistive devices they used, the difficulties they faced despite these devices, and their recommendations for a future visual prosthesis. 

## 2. Materials and Methods

In collaboration with two local retinitis pigmentosa patients with bare light perception vision in the better-seeing eye, we developed a 30-question survey (available in the Supplementary Materials), which was subsequently converted to an online format utilizing Survey Monkey. This survey was tested to ensure accessibility and ease of navigation by visually impaired patients using the Job Access With Speech (JAWS) screen reader or cell phone voice over accessibility features. A recruitment letter was sent to patients in the IRD clinic at the Bascom Palmer Eye Institute. In addition, through collaboration with the society leadership, an email invite was sent to individuals registered in the Foundation Fighting Blindness My Retina Tracker Program as well as in the Choroideremia Research Foundation database. The letter stated that a sighted individual could help them, if needed, to complete the survey.

We invited adult patients, at least 18-years of age, with VAs of 20/400 to no light perception in the better-seeing eye (only able to see the big “E” on the vision chart and worse in the better-seeing eye). Respondents were asked not to reveal any personal identifiable information. The first question of the survey was designed to determine whether the patient’s level of vision qualified them for the survey, and only those who answered the first question consistently with our recruitment goal were able to proceed with the rest of the survey. The second question asked the patient to specifically define their vision level in the better-seeing eye. To increase ease of completing the survey, respondents were allowed to skip questions. 

The responses were stratified by VA in the better-seeing eye as reported by the patient: 20/400, Finger Counting (CF), Hand Motion (HM), Light Perception (LP), and No Light Perception (NLP). Patients who have had an Argus II implant, all in the LP or NLP group, were sub-analyzed in a separate group since they had experience with an existing retinal prosthesis. Statistical analyses were performed using descriptive statistics provided by the Survey Monkey software. In SPSS (IBM, version 26), 2 × 2 contingency tables were generated through the CROSSTABS function, and Fisher’s exact tests were conducted. The Cochran–Armitage Trend test was performed to analyze the associations between VA and the devices used and limitations in activities. Statistically significant results were identified by corresponding two-sided *p*-values < 0.05.

The Institutional Review Board of the University of Miami School of Medicine Medical Sciences Subcommittee for the Protection of Human Subjects approved the current study (IRB ID 20201551), which adhered to the tenets of the Declaration of Helsinki. Consent was waived due to the anonymous nature of the survey.

## 3. Results

A total of 121 respondents qualified for the survey, indicated their visual acuity, and completed the survey. Several participants skipped questions, thus the total number of responses for each question varied. The participants’ characteristics are displayed in Table 1.

### 3.1. Activities of Daily Living to Be Improved and Assistive Devices Currently Used

Among the 103 respondents, reading (54.4%), navigating/exercising/traveling (51.5%), cooking (39.8%), using electronic devices (35.9%), and completing household chores/repairs/cleaning (35.9%) were most commonly listed as limitations experienced without the use of assistive devices (Table 2). The most common limitations despite the use of their assistive devices included the same activities. 

The assistive devices used for reading and mobility/orientation are listed in Table 2. The most commonly used devices were cellular phones/tablets for reading (63.6%) as well as a sighted guide (75.0%) and a cane (71.4%) for mobility. Comparing the VA groups (20/400, CF, HM, LP, and NLP), patients with worse VA more frequently used Braille for reading, (4.5%, 16.7%, 11.5%, 23.5%, and 45.5%, respectively, *p* = 0.0080) and less frequently used a magnifier glass (50.0%, 33.3%, 19.2%, 5.9%, and 0.0%, respectively, *p* < 0.001) and CCTV (45.5%, 41.7%, 26.9%, 17.6%, and 9.1%, respectively, *p* = 0.0054). For mobility and orientation, worse VA was associated with increased use of a cane (36.4%, 75.0%, 84.6%, 72.2%, and 90.9% respectively, *p* = 0.0014). Similarly, the use of a sighted guide increased from 59.1% in the 20/400 group to over 80% in the HM, LP, and NLP groups (*p* = 0.0086). Compared to respondents with just central vision loss, respondents with both central and peripheral vision loss more frequently reported utilization of a cane (*p* = 0.014) and the assistance of a sighted person (*p* = 0.0045) for mobility. 

Compared to younger respondents (18 to 64 years), older respondents (≥65 years) were more likely to use a guide dog for mobility and orientation (8.2% younger group, 29.7% older group, *p* = 0.0051). Older adults more frequently used nothing for reading (0% younger group, 8.3% older group, *p* = 0.035), while younger adults more frequently used computer screen readers (63.9% younger group, 44.4% older group, *p* = 0.065). A significantly greater frequency of young adults reported that they were bothered by their inability to read compared to older adults (55.8% younger group, 33.3% older group, *p* = 0.030).

Having medical insurance did not reveal a statistical difference in the likelihood of owning expensive assistive devices such as most technological devices.

### 3.2. Recommendations for Designing a Future Artificial Vision Prosthetic Device

Respondents were asked to choose five statements among a list of statements that reflected the activities that would be most significant to them if they were to design a bionic eye or prosthetic device to help them outside and inside, respectively. For help outdoors, the three most common responses were a prosthetic device that would help them avoid bumping into things when walking (71.4%), to read labels at the grocery store or packages (69.5%), and identify objects close by (64.8%) (Table 3). For help indoors, patients most frequently desired a device that would allow them to recognize faces (61.5%) and see small objects more clearly (53.8%) (Table 3).

Among the 101 patients who answered the question “If artificial vision could only restore one function of normal vision, please rank the five abilities below”, navigating independently in unfamiliar areas was ranked the highest priority for restoration (Table 3). Individuals in the 20/400 and CF groups ranked reading normal text in print and on devices the highest, while those in the HM, LP, NLP, and Argus groups ranked navigating independently in unfamiliar areas the highest. 

In order to agree to surgery for artificial vision, 101 survey respondents most frequently selected that they would need to have confidence that the surgery could at least allow them to see enough facial details to identify a person (43.6%) or enough to avoid an obstacle while walking (38.6%), well above the other choices listed (Table 3). 

Only 23 out of 101 (22.8%) respondents said that they would agree to have shape-based vision (Table 3). Interestingly, three out of four (75%) Argus recipients who responded to the question said that they would agree to have shape-based vision. When asked whether they would accept a narrow field artificial vision, 71 out of 99 (71.7%) answered yes (Table 3).

### 3.3. Suggested Improvements for Argus II Prosthesis among Argus Recipients

When asked to suggest improvements for the Argus^®^ II implant, Argus II recipients most frequently suggested technology that recognized and announced objects in front of them (*n* = 2), cut out busy flashing backgrounds (*n* = 2), and increased the ability to avoid objects and obstacles (*n* = 2) (Table 4).

## 4. Discussion

The aim of the present study was to assess the ADLs, challenges, and current assistive devices used by patients with IRDs and to determine their suggestions for future artificial vision prostheses. Our survey revealed that IRD patients with severe visual impairment and blindness continue to have difficulty with ADLs despite the assistive devices they currently use. Navigating with robust obstacle avoidance, reading normal text, and recognizing facial features were ranked the top priorities for designing a new prosthetic device. Shape-based vision would not be acceptable to the majority of patients. 

Various IRDs can progress to severe visual impairment and even loss of light perception. Until new treatments are developed that can reverse or prevent visual loss, patients with end-stage IRDs must rely on various assistive devices for help with ADLs. Our survey revealed that most ultra-low vision patients with IRDs rely on a sighted guide and a cane for mobility, and only approximately one-third used a technological or electronic device such as an iPhone, Trekker, tablet, Apple Watch, IrisVision, and Eye-Pal Rol. For reading purposes, cellular phone/tablet applications and accessibility features, computer screen readers, and the assistance of a sighted person were used by the majority of the respondents. Unfortunately, low vision evaluation and devices are not commonly covered by insurances in the U.S. including Medicare and stand-alone vision plans. However, legislative initiatives have been introduced to change this and many state level programs are beginning to provide assistance. 

Despite the currently available devices, patients continue to face challenges in performing ADLs, namely, navigating, cooking, using electronic devices, reading, and completing household chores. In the mid-2000s, ophthalmology entered a new era of prosthetic vision. An epiretinal prosthesis, the Argus^®^ II Retinal Prosthesis was approved in Europe in 2011, the USA in 2013, Canada in 2015, and Asia in 2017. It consists of a digital camera mounted on eyeglasses, which transmits wirelessly to an episcleral implant connected to a 60-microelectrode array attached to the inner surface of the retina [5,6]. The electrical impulses stimulate residual functioning ganglion cells, and the signal travels to the brain to produce artificial pixelated vision. More than 300 Argus^®^ II implants were implanted worldwide before the company lost funding and closed its doors during the COVID-19 pandemic. Other retinal prostheses have been developed such as Alpha IMS and Alpha AMS (both from Retina Implant AG, Reutlingen, Germany), but are no longer marketed due to their high cost and low utilization [4]. 

Due to current technology limitations, prosthetic vision generated by these devices is limited, and can be imagined by sighted individuals as flickering pixelated lights generated by brighter objects. Argus II recipients perform better with the system ON than system OFF on computer-based tasks and in some real-life tasks such as black and white sock sorting, following a sidewalk edge, and detecting the direction of a person walking in a laboratory setting [7,8]. Patients must undergo training in order to learn to interpret these lights [13]. Our local Argus II patients reported difficulty detecting edges and objects due to an excessive amount of artificial light, busy flashing light background, and flashing edges appearing simultaneously and extinguishing too quickly, making navigation and mobility outside challenging in certain ambient light conditions. The Argus II recipients suggested improvements to the current Argus artificial vision by increasing the ability to detect movement, adding technology capable of recognizing and announcing objects in front of them, and cutting out the busy flashing light background to facilitate navigation and the identification of objects. 

Two-thirds of all respondents would agree to artificial vision if it were very narrow and would be willing to scan the environment either with their eyes or with their head. In our experience, low vision specialists regard the central 20 degrees of field as the most critical for orientation and mobility and train patients to use canes and tactile and scanning techniques to augment the field. Notably, the Argus II prosthesis, which covers the macula, generates artificial vision of approximately 20 degrees [13]. When asked to suggest activities toward which a future prosthetic device should be targeted, the respondents chose navigation with robust obstacle avoidance, reading, and recognizing facial features and small objects as their top priorities. When asked to choose among the abilities provided by the current prosthetic technology that would be enough to undergo surgery for visual prosthesis, the most highly ranked features were facial recognition and the avoidance of obstacles while walking. Unfortunately, none of the current artificial vision prostheses have enough resolution to allow for normal reading. This may be improved by the incorporation of a scanner that reads to the patient, as suggested by the Argus respondents. We hope that the insights and recommendations of the ultra-low vision patients in this study will be helpful to the scientists developing new visual prosthetic technology worldwide, and that eventually, high-resolution prosthetic devices allowing patients to read normal text will become available. 

Limitations of the study include the anonymous nature of the survey, which did not allow for verification of the VA level, missing values to certain questions, and a small sample size of Argus patients. The online nature of the study excluded those patients who did not have access to email and technology to fill out the survey, thus reflecting the experiences of a portion of the ultra-low vision IRD community. The study did not include children given the feasibility and IRB constraints. As respondents were allowed to skip questions they did not wish to answer, incomplete questionnaires were included in our analysis and our study was subject to non-response bias. Patients with better VAs and those who experience fewer limitations in their activities of daily living, for instance, may have been less likely to respond to questions about the current limitations and recommendations for future prosthetic devices. As a result, our findings may disproportionately represent the population that continues to experience limitations in activities of daily living to be targeted for improvement with future prostheses. In addition, there were few subjects over the age of 80 years, a low percentage of African-American patients and other minorities, and slightly more males than females. 

In summary, despite the currently available non-invasive assistive devices, IRD patients with severe visual impairment and blindness continue to have difficulties with ADLs and frequently rely on another person for assistance. Navigation, reading, and recognizing facial features are ranked as top priorities when conceptualizing a new visual prosthesis. There is a continued need to develop higher resolution, voice-over, and versatile assistive technology as well as improve access to low vision services to increase the independence of patients with ultra-low vision. 

## Figures and Tables

**Table 1:** Characteristics of the study participants.

Variable	All Respondents	
	*n*	%
Visual Acuity (*n* = 121)		
20/400	23	19.0%
Counting Fingers (CF)	14	11.6%
Hand Motion (HM)	26	21.5%
Light Perception (LP)	41	33.9%
No Light Perception (NLP)	12	9.9%
Argus Recipient	5	4.1%
Length of Visual Impairment (*n* = 121)		
Less than 1 year	9	7.4%
1–10 years	53	43.8%
More than 10 years	59	48.8%
Gender (*n* = 117)		
Male	70	59.8%
Female	47	40.2%
Age (*n* = 116)		
18–34 years	15	12.9%
35–64 years	62	53.4%
65–80 years	37	31.9%
Over 80 years	2	1.7%
Race/Ethnicity (*n* = 118)		
White	88	74.6%
Black or African American	5	4.2%
Latino, Hispanic, or Spanish origin	15	12.7%
Asian	6	5.1%
American Indian or Alaska Native	1	0.8%
Middle Eastern	2	1.7%
Other	1	0.8%
Highest Education Completed (*n* = 114)		
Some High School	9	7.9%
High School Graduate	24	21.1%
Associate Degree (or some college)	25	21.9%
Bachelor’s Degree	31	27.2%
Master’s Degree	18	15.8%
Doctorate/PhD or higher	4	3.5%
Trade School	3	2.6%
Primary Country of Residence (*n* = 116)		
United States of America	114	98.3%
Canada	1	0.9%
European Country	1	0.9%
Geographic Area of Residence (*n* = 115)		
Rural	30	26.1%
Suburban	58	50.4%
Urban	27	23.5%
Current Vision (*n* = 114)		
Loss of central vision only	16	14.0%
Loss of central and side vision	98	86.0%
Inherited Retinal Disease (IRD) (*n* = 105)		
Retinitis Pigmentosa	54	51.4%
Cone-Rod Dystrophy	10	9.5%
Stargardt Disease	10	9.5%
Choroideremia	10	9.5%
Leber Congenital Amaurosis	4	3.8%
Other	12	11.4%
Multiple diseases	5	4.8%
Assistance with Daily Activities (*n* = 116)		
Has a person who regularly helps	57	49.1%
Has a person who occasionally helps	44	37.9%
Does not have anyone who helps	15	12.9%
Employment Status (*n* = 109)		
Employed	27	24.8%
Unemployed	82	75.2%
Volunteering Status (*n* = 110)		
Volunteer	20	18.2%
Non-volunteer	90	81.8%

**Table 2:** Limitations in activities of daily living to be targeted for improvement and the assistive devices currently used.

Variable	All Respondents	
	*n*	%
Activities INSIDE OR OUTSIDE OF HOME due to poor vision without any assistive devices that respondents report as most important to improve or regain (*n* = 103)		
Reading	56	54.4%
Navigating/Exercising/Traveling	53	51.5%
Cooking	41	39.8%
Using computer or phone/Watching TV	37	35.9%
Household Chores/Repairs/Cleaning	37	35.9%
Yard work	24	23.3%
Hobbies (e.g., doing crafts, playing video games)	23	22.3%
Driving	20	19.4%
Shopping	20	19.4%
Selecting and matching clothing	13	12.6%
Locating/identifying objects and people	12	11.7%
Recognizing faces and facial expressions	11	10.7%
Laundry	10	9.7%
Other (managing finances, medical care, professional and academic pursuits, etc.)	53	51.5%
Assistive Devices Used for Reading (*n* = 110)		
Cellular phone/tablet apps and accessibility features	70	63.6%
Computer screen reader	63	57.3%
Another person	60	54.5%
Scanner or hand-held reader	39	35.5%
CCTV magnifiers	29	26.4%
Magnifying glass	22	20.0%
Braille	20	18.2%
Other (OrCAM device, computer with large font, digital talking book player, wearable magnifier, electronic magnifier)	18	16.4%
None	3	2.7%
Assistive Devices Used for Mobility and Orientation (*n* = 112)		
Another person to assist	84	75.0%
Cane	80	71.4%
Technological device *	37	33.0%
Guide dog	17	15.2%
Aira service	9	8.0%
None	5	4.5%
Argus II retinal prosthesis	2	1.8%

* Technological devices used for mobility and orientation included iPhone, Trekker. GPS-based guiding device that announces streets, intersection, and points of interest), tablets, Apple Watch, IrisVision headset (wearable magnifier with voice controls and cellular connectivity), and Eye-Pal Rol (portable scanner and reader).

**Table 3:** Designing a future prosthetic device to assist with daily activities.

Variable	All Respondents	
	*n*	%
If you could design a bionic eye or prosthetic device to help you OUTDOORS, which activities would be more significant to you (respondents chose 5 statements from the list of 10 choices)? (*n* = 105)		
It would help avoid bumping into things when walking	75	71.4%
It would help me read labels at the grocery store or packages	73	69.5%
It would help me identify objects close by	68	64.8%
It would help me judge how far objects are away from me	63	60.0%
It would help me read street signs and store names	62	59.0%
It would help me detect moving objects such as cars	62	59.0%
It would help me cross street at a traffic light	60	57.1%
It would help me identify objects far away	56	53.3%
It would help me walk straight in an open space	45	42.9%
It would give me more side vision (wider field of vision)	36	34.3%
If you could design a bionic eye or prosthetic device to help you INSIDE, which activities would be more significant to you (respondents chose 5 statements from the list of 18 choices)? (*n* = 104)		
It would help me recognize faces	64	61.5%
It would help me see small objects more clearly	56	53.8%
It would help me use a computer without a screen reader	50	48.1%
It would help me watch TV or movies	50	48.1%
It would help me with household tasks such as cleaning, laundry	48	46.2%
It would help me prepare meals or fix a snack	45	43.3%
It would help me read single letters and numbers	44	42.3%
It would help me sign my name	39	37.5%
It would help me identify money	39	37.5%
It would help me judge how far objects are away from me	37	35.6%
It would help me locate doors	37	35.6%
It would help me see colors when matching clothes for example	36	34.6%
It would help me locate and track silent people	30	28.8%
It would give me more side vision (wider field of vision)	24	23.1%
It would help me find clear glass doors	21	20.2%
It would help me with hand-eye coordination	20	19.2%
It would help me play video games	12	11.5%
It would help me identify sizes of objects	11	10.6%
If artificial vision could only restore one function of normal vision, please rank the five abilities below from 1 through 5, with “5” being “I definitely want restored”, and “1” being “I don’t need this restored” (*n* = 101)	Median (Range)	
Navigate independently in unfamiliar areas	3.6 (1–5)	
Read normal text, in print and on devices	3.3 (1–5)	
Able to see and recognize individual faces in detail	3.2 (1–5)	
Watch TV or movies and use a computer without a screen reader	3.0 (1–5)	
See colors and patterns in clothing, nature, and art	2.4 (1–5)	
“In order to agree to surgery for artificial vision, I would have to have confidence that the surgery can at least allow me to see…”		
(respondents chose 1 statement from the list of 7 choices) (*n* = 101)		
Enough facial detail to identify a person	44	43.6%
Enough to avoid an obstacle while walking	39	38.6%
Enough to know that a person is present, even if I cannot identify them	8	7.9%
Enough to locate a cup or utensil on a table	6	5.9%
Enough to tell if a sock is black or white	3	3.0%
Enough to know if a light is on or off	1	1.0%
Enough to find a door	0	0.0%
On a scale from 1 to 5, how important is the ability for you to see color as opposed to black and white with artificial vision. (“5” is “Very important”, and “1” is “Not very important”) (*n* = 103)	Mean (Range) 3.5 (1–5)	
Would you agree to have shape-based vision? (*n* = 101)		
No	78	77.2%
Yes	23	22.8%
Artificial vision may be limited in how much you can see at one time, it may appear as if you are looking through a straw, and this may require scanning around with your head (pointing your head in different directions) or scanning with your eyes (moving eyes back and forth) to see. Knowing this limitation, would you agree to have the artificial vision procedure? (*n* = 99)		
No	28	28.3%
Yes, I am ok with scanning with my head	35	35.3%
Yes, I prefer scanning with my eyes	36	36.4%

**Table 4:** Suggested improvements for the Argus II prosthesis among the Argus recipients.

Variable	All Respondents	
	*n*	%
If you have had an Argus II implant and are familiar with the artificial vision it provides, what one improvement would you suggest that is most significant to you? (*n* = 5)		
Add technology able to recognize objects and tell me what they are	2	40.0%
Increase ability to avoid objects and obstacles	1	20.0%
Improve inversion of image from black-to-white and white-to-black	1	20.0%
Make glasses more comfortable	1	20.0%
If you have had an Argus II implant and are familiar with the artificial vision it provides, what additional three improvements would you suggest? (*n* = 3)		
Cut out the busy flashing background	2	66.7%
Increase ability to detect movement	2	66.7%
Make the field of artificial vision wider	1	33.3%
Add technology able to recognize objects and tell me what they are	1	33.3%
Improve detection of distances	1	33.3%
Increase ability to avoid objects and obstacles	1	33.3%
Increase definition of the object’s shapes	1	33.3%

## Data Availability

The data presented in this article are available upon request from the authors. Individual survey monkey responses are no longer available due to accounts being inactivated after payment period expired.

## Supplementary Materials

The following supporting information can be downloaded at: https://www.mdpi.com/article/10.3390/jcm12165283/s1, Supplemental Digital Content 1.pdf (this is the questionnaire administered).
