# Supplementary material for: Current Assistive Devices Usage and Recommendations for a Future Artificial Vision Prosthesis among Patients with Severe Visual Impairment Due to Inherited Retinal Diseases
Source: J Clin Med. 2023 Aug 14;12(16):5283. doi: 10.3390/jcm12165283 (PMC10455651; doi:10.3390/jcm12165283)
Supplement: Supplementary file 1 [file jcm-12-05283-s001.zip › jcm-2535990-supplementary.pdf]

This survey is 30 questions long and should take approximately 30 minutes to complete.

The purpose of this survey is to learn about specific challenges in daily activities of individuals with low vision and to hear their suggestions for a new prosthetic device or bionic eye. You are invited to participate in this survey because you have a retinal degenerative condition and have low vision. If you are only able to see the "big E" on the chart or worse with your better eye, you qualify for the study.

All of your answers will be strictly autonomous and will not be traced back to your identity.

Only people who are directly involved with this project will have access to your answers.
When the project is finished and results are reported, no individual will be identified in any way.

Your participation is voluntary. If you agree to participate, please proceed by answering the questions below. You can decline to participate, and you can stop this survey at any time, if you wish to do so, without any negative consequences to you.

By you answering the survey questions below, this means you consent to participate in this research project.

1. **What is your vision in the better-seeing eye with your glasses or contact lenses if any [select one answer below]?**
A. ☐ I see the big E and other smaller letters on the vision chart → Thank you for taking the time to begin the survey. Your vision is above the level being studied in this survey.
B. ☐ I only see the big E on the chart → please proceed with the survey
C. ☐ I do not even see the big E on the chart → please proceed with the survey

2. **Please define your vision in the better-seeing eye [please check all that apply]?**
A. ☐ I can see the big E on the chart and no other letters
B. ☐ I can count how many fingers someone else holds in front of me
C. ☐ I can see the direction in which someone moves their hand in front of me
D. ☐ I can see if lights are on or off
E. ☐ I cannot tell when the room lights are on or off

3. **How long has your vision been at this level [select one answer below]**?
A. ☐ less than 1 year
B. ☐ 1-10 years
C. ☐ more than 10 years

4. **What is your age [select one answer below]?**
A. ☐ 18-34 years
B. ☐ 35-64 years
C. ☐ 65-80 years
D. ☐ over 80 years

5. **Do you identify yourself as a male or female?**
A. ☐ Female
B. ☐ Male
C. ☐ Other

6. **What is your race or origin [select the statement below that best describes your origin or race]?**
A. ☐ White
B. ☐ Black or African-American
C. ☐ Latino, Hispanic, or Spanish origin

D. ☐ Asian
E. ☐ American Indian or Alaska Native
F. ☐ Native Hawaiian or Pacific Islander
G. ☐ Middle Eastern
H. ☐ Some other race or origin - list race or origin

7. **What is the highest degree or level of education you have completed?**
A. ☐ Some High School (secondary school)
B. ☐ High School Graduate (secondary school)
C. ☐ Associate Degree (or some college)
D. ☐ Bachelor's Degree
E. ☐ Master's Degree
F. ☐ Doctorate/Ph.D. or higher
G. ☐ Trade School

8. In what country do you live most of the year?
A. ☐ USA
B. ☐ Canada
C. ☐ A European country
D. ☐ A Central or South American country
E. ☐ An African country
F. ☐ An Asian country
G. ☐ Australia
H. ☐ New Zealand
I. ☐ Other- please list

9 In what setting to you live most of the year?
A. ☐ Rural
B. ☐ Suburban
C. ☐ Urban

10. Do you have someone who assists you with daily activities? Select the statement that applies to you.
A. ☐ I do not have anyone who helps me with daily activities.
B. ☐ I either live with or have a person who OCCASIONALLY helps me with daily activities.
C. ☐ I either live with or have a person who REGULARLY helps me with daily activities.

11. Which statement below best describes your current vision?
A. ☐ Loss of central vision only (big smudge in the middle of my vision that does not allow me to see fine details, but my side vision is good)
B. ☐ My entire vision is poor with loss of central and side vision (peripheral)
C. ☐ Other, please describe

12. Please list the eye condition and the disease-causing gene (if you know it) that has led to your loss of vision:
☐

13. Do you have hearing loss? Please select the statement below that best applies to you.
A. ☐ I do not have hearing loss
B. ☐ I have hearing loss and it is part of my genetic disorder
C. ☐ I have hearing loss but it is NOT a part of my genetic disorder
D. ☐ I have hearing loss but I am not sure if it is related to my genetic disorder

14. Do you receive any government or insurance financial support to help you pay for the devices you use for orientation and mobility or reading [please select all that apply]?

A. ☐ No

B. ☐ Yes, Medicare pays for me

C. ☐ Yes, Medicaid pays for me

D. ☐ Yes, private insurance pays, please list the name of your insurance: ____________

E. ☐ Yes, another source pays, please list: ________________

15. Please select from the list below any tools you use for mobility and orientation. Please select all that apply.

A. ☐ Cane

B. ☐ Guide dog

C. ☐ Another person to assist me

D. ☐ Technological device, please specify ___________________________

E. ☐ Prosthetic device, please specify___________________________

F. ☐ Aira service where a live remotely located person provides assistance

G. ☐ Other, please list_________________________

H. ☐ None

16. Please select from the list below all you use to assist you in reading [select all that apply].

A. ☐ Braille

B. ☐ Hand-held reader

C. ☐ CCTV magnifiers

D. ☐ Another person helps me read

E. ☐ Computer screen reader like JAWS

F. ☐ OrCAM device

G. ☐ Cellular phone apps

H. ☐ Magnifying glass

I. ☐ Scanner

J. ☐ Other, please list_____________________

K. ☐ None

17. If you are currently employed, what do you do in your job? You do not need to name the company you work for if you prefer not to, just tell us about your job title and job responsibilities. If you are not currently employed, please state.

A. ☐ I am not currently employed

B. ☐ I am currently employed and my job title and responsibilities are____

18. If you currently volunteer, please describe where you volunteer and in what capacity. If you currently do not volunteer, please state.

A. ☐ I do not volunteer

B. ☐ I currently volunteer, please describe where and in what capacity____

19. Limitation of Activities INSIDE OR OUTSIDE OF YOUR Home Due to Poor Vision WITHOUT ANY ASSISTIVE DEVICES

We are interested to find out what limitations you would or currently have without the help of any assistive devices or another person helping you with daily activities at your home. Without any of your assistive devices or another person helping you, list UP TO five (5) activities or tasks around your home that would be most important for you to improve or regain:

A. ☐ activity or task one:

B. ☐ activity or task two:

C. ☐ activity or task three:

D. ☐ activity or task four:

E. ☐ activity or task five:

20. **Limitation of Activities INSIDE OR OUTSIDE OF YOUR Home Due to Poor Vision DESPITE YOUR CURRENT ASSISTIVE DEVICES**

Now we are interested to find out what limitations you still have at your home DESPITE all your assistive devices. Please list UP TO five (5) activities or tasks around your home that you wish you could further improve or regain when using all your current assistive devices:

A. ☐ activity or task one:

B. ☐ activity or task two:

C. ☐ activity or task three:

D. ☐ activity or task four:

E. ☐ activity or task five:

21. If you could design a bionic eye or prosthetic device to help you OUTDOORS, which activities would be more significant to you?
Please choose 5 statements from the below list of 8 statements that are most significant to your life.

A. ☐ It would help me cross street at a traffic light

B. ☐ It would help me walk straight in an open space

C. ☐ It would help avoid bumping into things when walking, like people and tree branches

D. ☐ It would help me judge how far objects are away from me (depth perception)

E. ☐ It would give me more side vision (wider field of vision)

F. ☐ It would help me detect moving objects such as cars

G. ☐ It would help me identify objects close by

H. ☐ It would help me identify objects far away

I. ☐ It would help me read labels at the grocery store or packages

J. ☐ It would help me read street signs and store names

22. If you could design a bionic eye or prosthetic device to help you INDOORS, which activities would be more significant to you?
Please choose 5 statements from the below list of 18 statements that are most significant to your life.

A. ☐ It would help me judge how far objects are away from me (depth perception)

B. ☐ It would give me more side vision (wider field of vision)

C. ☐ It would help me see small objects more clearly

D ☐ It would help me locate and track silent people

E. ☐ It would help me locate doors

F. ☐ It would help me find clear glass doors

G. ☐ It would help me with hand-eye coordination

H. ☐ It would help me identify sizes of objects

I. ☐ It would help me read single letters and numbers

J. ☐ It would help me play video games

K. ☐ It would help me sign my name

L. ☐ It would help me prepare meals or fix a snack

M. ☐ It would help me with household tasks such as cleaning, laundry

N. ☐ It would help me identify money

O. ☐ It would help me recognize faces

P. ☐ It would help me watch TV or movies

Q. ☐ It would help me use a computer without a screen reader

R. ☐ It would help me see colors when matching clothes for example

23. If artificial vision could only restore one function of normal vision, please rank the five abilities below from 1 thru 5, with "5" being "I definitely want restored", and "1" being "I don't need this restored" . Give each statement a unique number from 1 to 5, and please do not use the same number more than once.

A. ☐ Able to see and recognize individual faces in detail.

B. ☐ Navigate independently in unfamiliar areas.

C. ☐ Read normal text, in print and on devices.

D. ☐ Watch TV or movies and use a computer without a screen reader.

E. ☐ See colors and patterns, in clothing, nature and art.

24. Artificial vision may provide varying levels of details that you can see.
Please complete the following sentence by choosing ONE statement that best reflects your opinion.
"In order to agree to surgery for artificial vision, I would have to have confidence that the surgery can at least allow me to see…"

A. ☐ enough facial detail to identify a person

B. ☐ enough to know that a person is present, even if I cannot identify them

C. ☐ enough to find a door

D. ☐ enough to know if a light is on or off

E. ☐ enough to locate a cup or utensil on a table

F. ☐ enough to avoid an obstacle while walking

G. ☐ enough to tell if a sock is black or white

25. On a scale from 1 to 5, how important is the ability for you to see color as opposed to black and white with artificial vision. ("5" is "Very important", and "1" is "Not very important") ☐

26. Artificial vision may be completely different from normal sight. If artificial vision uses only shapes or patterns to represent what you see, such as a person appearing like a bar, a car appearing like a rectangle, or a building appearing like a triangle, would you agree to have a procedure to have shaped based vision?

A. ☐ yes

B. ☐ no

27. Artificial vision may be limited in how much you can see at one time, it may appear as if you are looking thru a straw, and this may require scanning around with your head

(pointing your head in different directions) or scanning with your eyes (moving eyes back and forth) to see. Knowing this limitation, would you agree to have the artificial vision procedure? Please choose the best answer below.

A. ☐ No I would not agree to “straw-like” artificial vision

B. ☐ Yes, I am ok scanning with my head

C. ☐ Yes, I prefer scanning with my eyes rather than with my head

28. Have you had an Argus II implanted in your eye?
If not, skip this question.
If you have had an Argus II implant and are familiar with the artificial vision it provides, what improvements would you suggest? Please choose ONE statement that is the most significant to you.

A. ☐ Make the field of artificial vision wider

B. ☐ Cut out the busy background (The device needs to focus on the main object and nothing else around)

C. ☐ Add technology able to recognize objects in front of me and tell me what they are

D. ☐ Improve detection of distances, to be able to tell what objects are close by and which ones are far away

E. ☐ Increase ability to avoid objects and obstacles

F. ☐ Increase ability to detect movement (cars for example)

G. ☐ Increase definition of the object’s shapes

H. ☐ Add technology to it so that it can read to me things like labels or menus

I. ☐ Other- use your own words

29. Now, please answer the previous question one more time by choosing three more statements that are important to you.
To clarify, if you have had an Argus II implant and are familiar with the artificial vision it provides, what three improvements would you suggest? Please choose THREE statements from the list below, but DO NOT choose the statement you already selected in the previous question.

If you have not had an Argus implant, skip to the next question.

A. ▢ Make the field of artificial vision wider

B. ▢ Cut out the busy background (The device needs to focus on the main object and nothing else around)

C. ▢ Add technology able to recognize objects in front of me and tell me what they are

D. ▢ Improve detection of distances, to be able to tell what objects are close by and which ones are far away

E. ▢ Increase ability to avoid objects and obstacles

F. ▢ Increase ability to detect movement (cars for example)

G. ▢ Increase definition of the object's shapes

H. ▢ Add technology to it so that it can read to me things like labels or menus

I. ▢ Other- use your own words

30. How would you describe yourself [select all that apply]?

A. ▢ I typically see things as "glass half full"

B. ▢ I typically see things as "glass half empty"

C. ▢ I am easy going

D. ▢ I am easily upset and annoyed by things or people

E. ▢ I am easily satisfied

F. ▢ It is difficult for me to be entirely satisfied and happy unless I perceive things as perfect

G. ▢ I am an optimist

H. ▢ I like to try new things and experiences

I. ▢ I am typically hesitant to try new things
